# Comprehensive Analysis of Acquired Genetic Variants and Their Prognostic Impact in Systemic Mastocytosis

**DOI:** 10.3390/cancers14102487

**Published:** 2022-05-18

**Authors:** Oscar González-López, Javier I. Muñoz-González, Alberto Orfao, Iván Álvarez-Twose, Andrés C. García-Montero

**Affiliations:** 1 Cancer Research Center (IBMCC, USAL/CSIC), Department of Medicine, Universidad de Salamanca, Biomedical Research Institute of Salamanca and Spanish Network on Mastocytosis (REMA), 37007 Salamanca, Spain; oscar_villa_93@usal.es (O.G.-L.); jmunogon@usal.es (J.I.M.-G.); orfao@usal.es (A.O.); 2 Centro de Investigación Biomédica en Red Cáncer (CIBERONC), 28029 Madrid, Spain; ivana@sescam.jccm.es; 3 Instituto de Estudios de Mastocitosis de Castilla La Mancha (CLMast, Virgen del Valle Hospital) and REMA, 45071 Toledo, Spain

**Keywords:** systemic mastocytosis, prognostic, mutations, *KIT*, D816V, *ASXL1*, *DNMT3A*, *EZH2*, *RUNX1*, *SRSF2*

## Abstract

**Simple Summary:**

Systemic mastocytosis (SM) is a clonal haematopoietic stem cell disease typically characterized by the expansion and accumulation of neoplastic mast cells carrying the activating *KIT* D816V as a driver mutation. Multilineage involvement of haematopoiesis by this *KIT* mutation, particularly in a multi-mutated context, also involving other genes (e.g., *SRSF2*, *ASXL1*, *DNMT3A*, *RUNX1*, *EZH2*, *CBL* and *NRAS*) found to be frequently mutated in other myeloid neoplasms, have recently emerged as a genetic background associated with malignant transformation of SM. Therefore, assessment of multilineage involvement of haematopoiesis by *KIT* D816V and additional mutations in genes known to be associated with the prognosis of SM have become of great help to identify good vs. poor-prognosis SM patients who could benefit from a closer follow-up and, eventually, also early cytoreductive treatment.

**Abstract:**

Systemic mastocytosis (SM) is a rare clonal haematopoietic stem cell disease in which activating *KIT* mutations (most commonly *KIT* D816V) are present in virtually every (>90%) adult patient at similar frequencies among non-advanced and advanced forms of SM. The *KIT* D816V mutation is considered the most common pathogenic driver of SM. Acquisition of this mutation early during haematopoiesis may cause multilineage involvement of haematopoiesis by *KIT* D816V, which has been associated with higher tumour burden and additional mutations in other genes, leading to an increased rate of transformation to advanced SM. Thus, among other mutations, alterations in around 30 genes that are also frequently mutated in other myeloid neoplasms have been reported in SM cases. From these genes, 12 (i.e., *ASXL1*, *CBL*, *DNMT3A*, *EZH2*, *JAK2*, *KRAS*, *NRAS*, *SF3B1*, *RUNX1*, *SF3B1*, *SRSF2*, *TET2*) have been recurrently reported to be mutated in SM. Because of all the above, assessment of multilineage involvement of haematopoiesis by the *KIT* D816V mutation, in the setting of multi-mutated haematopoiesis as revealed by a limited panel of genes (i.e., *ASXL1*, *CBL*, *DNMT3A*, *EZH2*, *NRAS*, *RUNX1* and *SRSF2*) and associated with a poorer patient outcome, has become of great help to identify SM patients at higher risk of disease progression and/or poor survival who could benefit from closer follow-up and eventually also early cytoreductive treatment.

## 1. Introduction

Systemic mastocytosis (SM) is a rare hematologic disease characterized by an abnormal expansion and accumulation of pathological mast cells (MCs) in skin and/or other several extracutaneous tissues such as bone marrow (BM) and the gastro-intestinal tract. Currently, SM is divided into five different diagnostic subtypes according to the World Health Organization (WHO) 2016 classification [1]. These include indolent SM (ISM), smouldering SM (SSM), aggressive SM (ASM), SM with associated haematological neoplasms (SM-AHN) and MC leukaemia (MCL). Additionally, the inclusion of two new subtypes of SM into the classification of the disease is currently under consideration: a variant of ISM known as BM mastocytosis (BMM) [2,3], which is characterized by a low BM MC burden in the absence of skin lesions, and a very rare (<5%) variant of mastocytosis, which shows tumour mast cells (MCs) with a well-differentiated morphology together with a CD25^−^ CD2^−^ immunophenotype and unique clinical, biological and molecular features, termed well-differentiated SM (WDSM) [4]. From a prognostic point of view, all these diagnostic subtypes of SM can be grouped into (i) non-advanced forms of SM (Non-AdvSM), which include BMM, ISM and SSM, typically characterized by a more stable and indolent course of the disease and a life expectancy similar or close to that of a sex- and age-matched population; and (ii) advanced SM (AdvSM) including ASM, SM-AHN and MCL, which typically display an adverse prognosis associated with a significantly shortened life expectancy requiring cytoreductive therapy [1]. Despite this, some ISM patients (<5%) can eventually evolve to SSM and AdvSM [5]. Conversely, a small proportion of AdvSM patients may also show a relatively stable disease course over years or even decades [6,7].

Currently, the aetiopathogenic mechanisms involved in malignant transformation of SM remain largely unknown. However, from an ontogenetic point of view, it is known that pathological MCs from the vast majority of patients (>90%) carries the D816V mutation in the *KIT* protooncogene [8,9] regardless of the diagnostic subtype of SM and its clinical course (e.g., Non-AdvSM or AdvSM). Despite this, multilineage involvement of hematopoietic cells other than MCs by *KIT* D816V, together with the existence of additional mutations in genes other than *KIT* (e.g., *SRSF2*, *ASXL1*, *RUNX1*, *EZH2*) have been demonstrated in recent years to mostly affect (but not only) AdvSM patients [10,11,12]. It is noteworthy that most of the latter mutations involve genes that are also recurrently mutated in other (myeloid) haematological malignancies [10,11,13], suggesting a tendency for their acquisition in haematopoietic cells that have an appropriate altered genetic background (i.e., *KIT* D816V-positive cells) that could favour malignant transformation and a more aggressive disease behaviour.

## 2. *KIT* Mutations in Systemic Mastocytosis

The *KIT* gene is a proto-oncogene encoding for a trans-membrane receptor (mast/stem cell growth factor receptor (KIT)) with tyrosine kinase (TK) activity located on the long arm of human chromosome 4 [14]. When the KIT ligand—stem cell growth factor (SCF)—binds to KIT, conformational changes occur that lead to dimerization of the receptor and its activation by autophosphorylation [15]. Of note, intracellular signalling triggered upon activation of the KIT receptor is key to the normal development of haematopoiesis and the survival of haematopoietic stem cells (HSC) [16]. Except for MCs and some natural killer (NK) cells, *KIT* is no longer expressed by other mature myeloid and lymphoid haematopoietic cells [17]. In MCs, *KIT* expression remains at high levels throughout maturation [18,19], playing a critical role in MC proliferation, differentiation and survival [15,20]. Therefore, the acquisition of mutations that could impair the normal function of KIT (e.g., activating *KIT* mutations) has pro-oncogenic effects associated with inhibition of apoptosis and increased MC proliferation and survival [21,22].

### 2.1. KIT D816V Mutation

The D816V mutation of *KIT* is located at exon 17 within the tyrosine kinase (TK) 2 domain of the *KIT* gene. This mutation causes constitutive activation of the KIT receptor in the absence of SCF binding and represents the most frequent genetic alteration in SM (>90% of adult SM patients) [9,15]. In fact, constitutive activation of KIT causes preferential differentiation of HSC toward cell lines regulated by *KIT* expression and signalling (mainly MCs and to a large extent also other myeloid lineages). The fact that MCs are the only haematopoietic cells that express *KIT* throughout their maturation [18,19] would explain why this *KIT*-activating mutation induces the expansion and accumulation of pathological MCs in different organs and tissues, as typically observed in SM and other *KIT*-mutated MC diseases [23]. Of note, the prevalence of the *KIT* D816V mutation is very similar among adult patients diagnosed with Non-AdvSM and AdvSM [9]. Therefore, the *KIT* D816V mutation is considered as a (specific) diagnostic marker of SM, regardless of the subtype of the disease, its presence being one of the four minor criteria required by WHO for the diagnosis of SM [1,24,25]. However, the presence of this mutation cannot explain by itself the wide spectrum of disease behaviour observed among SM patients, ranging from stable and even pauci-symptomatic to progressive and even highly-aggressive disease [5].

### 2.2. Other KIT Mutations

Overall, *KIT* mutations other than *KIT* D816V can be found in up to 4–5% adults and one third of children with mastocytosis [9]. In adults, these mutations are mostly located at codons 814–822 within exon 17 [9,26,27,28,29,30,31], including several mutant variants at codon 816 [9,32,33,34,35,36,37,38,39,40,41,42,43,44,45,46] (Table 1). *KIT* mutations located outside exon 17 include rare mutations that mostly affect exons 2 [29], 5 [40], 7–11 [29,32,40,46,47,48,49,50,51,52,53,54,55,56,57,58], 13 [29,59] and 18 [29]. Of note, most mutations other than *KIT* D816V correspond to isolated cases of SM-AHN, MCL or WDSM (Table 1). Interestingly, MCL patients with *KIT* mutations other than D816V often lack additional somatic high-risk mutations [46]. Although the vast majority of *KIT* mutations defined above are acquired (somatic) genetic variants, a few mutations typically located in exons 8 to 10 of *KIT* (e.g., delD419 [60], S451C [61], K509I [62,63] or F522C [54]) correspond to germinal mutations that frequently show a familial aggregation pattern.

From a clinical point of view, the exact location of the mutations in the *KIT* gene is of great relevance, since those mutations that occur within the transmembrane or juxtamembrane domains of the *KIT* gene (exons 9–11) induce spontaneous receptor dimerization, making pathological MCs sensitive to conventional TK inhibitor therapies (e.g., imatinib) [52,53,54,55,62,64], while *KIT* mutations involving the catalytic domain (exons 13–18) cause a conformational change of the protein, which confers intrinsic resistance to imatinib and other TK inhibitors commonly used to treat other human tumours [65,66].

## 3. Clonal Haematopoiesis in Systemic Mastocytosis

SM is considered a clonal HSC disease characterized by the expansion and accumulation of neoplastic MCs [69,70,71]. As a neoplasm involving the HSC compartment, the *KIT* D816V (and other *KIT*) mutations can be found in both neoplastic MCs and CD34^+^ BM HSC, as well as in other myeloids (e.g., neutrophils [9,72,73,74], monocytes [9,70,72,73,74], basophils [70,72,74] and/or eosinophils [9,74]) and/or lymphoid (e.g., T and B lymphocytes [9,70,73,74]) cells. In such cases presenting multilineage involvement of haematopoiesis, clonal myeloid (MM) or myeloid plus lymphoid (MML) cells are found, which derive from the expansion and differentiation of D816V-mutated HSCs to different myeloid and/or lymphoid cell lineages [5,75]. Moreover, *KIT* D816V-mutated BM mesenchymal stem cells (MSCs) are also frequently detected in MML-mutated cases [36,76,77]. Overall, multilineage involvement of haematopoiesis by the *KIT* D816V mutation is found in virtually all ASM and SSM patients, in around one third of ISM cases and in a small proportion (≤10%) of BMM patients [9,78]. In SM-AHN, the frequency of patients that show a multilineage *KIT* D816V mutation may vary significantly [36] depending on the specific subtypes of SM and AHN [9]. Thus, *KIT* D816V-mutated AHN cells have been found in 89% of SM associated with chronic myelomonocytic leukaemia (SM-CMML), while this would only occur in 20% of SM associated with myeloproliferative neoplasms (MPN) and 30% of SM associated with acute myeloblastic leukaemia (AML); in turn, the *KIT* mutation is almost systematically restricted to the MC compartment in patients with SM associated with lymphoid neoplasms [36].

## 4. Mutations in Genes Other Than *KIT*

Emergence of the *KIT* D816V mutation in an HSC during the development of haematopoietic cells would potentially lead to multilineage involvement of haematopoiesis [77]. This would favour the expansion of neoplastic MCs and an increasing tumour burden; in addition, it might also lead to an increased genomic instability that may facilitate acquisition and accumulation of additional genetic alterations (Table 2 and Table S1) in the *KIT*-mutated or unmutated HSC and contribute to the malignant transformation of the disease via distinct molecular mechanisms, e.g., activation/repression of anti-/pro-apoptotic mechanisms [79].

**Figure 1:**
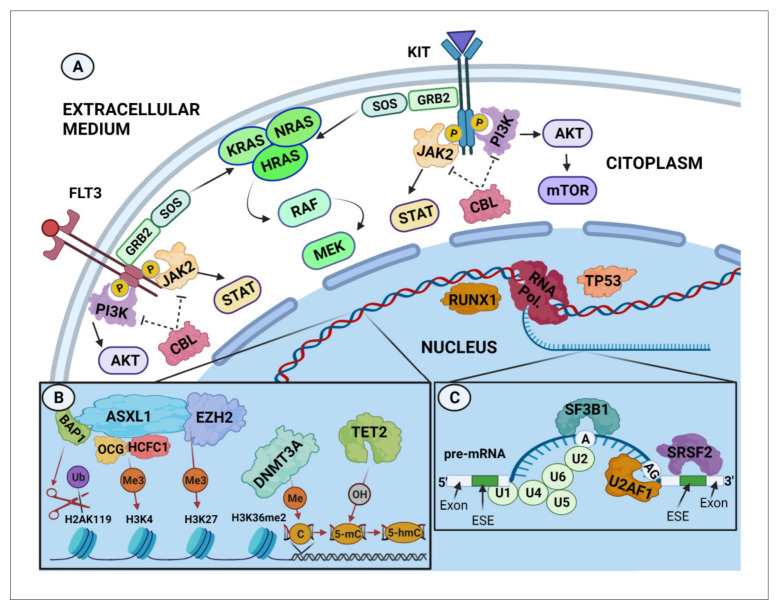
Genes recurrently mutated in systemic mastocytosis categorized by cellular functions. (**A**) Signal transduction and transcription regulation. Extracellular signals are received and transmitted effectively into the cell by activation of cell-surface receptors such as tyrosine kinase receptors TKR (e.g., FLT3 or KIT), resulting in the activation of intracellular signalling cascades, including the MAPK (i.e., RAS), STATs (i.e., JAK2) and PI3K pathways, which promote cell proliferation, survival and apoptosis by inducing gene transcription and/or DNA epigenetic modifications [98]. Activation/repression of these pathways require appropriate regulation of the activity and/or quantity of specific proteins. As an example, CBL proteins negatively regulate TKR (e.g., FLT3, KIT) and non-TKR (e.g., PI3K, JAK2) proteins through their ubiquitination and proteasomal degradation [99]. (**B**) Epigenetic regulation. ASXL1 and *EZH2* are members of the Polycomb group (PcG) of proteins, which are considered necessary to disrupt chromatin compaction in localized areas by activating/repressing specific histone markers. *EZH2* is involved in transferring methyl groups to histone H3 lysine 27 (H3K27), whereas ASXL1, associated with BAP1, is involved in de-ubiquitinating mono-ubiquitinated histone H2AK119 and, when associated with the OGT/HCFC1 complex, in the methylation (Me3) of H3K4 [100]. The DNMT3A protein is recruited by the histone mark H3K36me2 [101] to be involved in the methylation of cytosines (5mC), whereas the TET protein family is involved in active demethylation through oxidation of 5mC to 5hmC [102]. Overall, this mechanism results in enhancing transcription of certain genes while repressing the transcription of other genes. (**C**) RNA splicing. At the pre-mRNA level, the SF3B1, SRSF2 and U2AF1 proteins cooperate with U1–U6 small nuclear ribonucleoproteins (sn-RNPs), forming the U2-dependent splicing complex that brings the two intronic ends together by attaching the two exons and removing the intron [103]. This process transforms the pre-mRNA into mRNA, which can be transduced into a protein by ribosomes. Abbreviations: A, branch site; AG, splice receptor site; BAP1, BRCA-1-associated protein 1; ESE, exonic splicing enhancer; 5mC, 5 methyl cytosine; H, histone; HCFC1, host cell factor C1; Me, methylation; OH, hydroxylation; OGT, O-linked *N*-acetylglucosamine (GlcNAc) transferase; pre-mRNA, precursor messenger RNA; PcG, polycomb group; RTKs, receptor tyrosine kinases; Ub, ubiquitin; U1-U6, small nuclear ribonucleoproteins (snRNPs). Created using BioRender.

In line with this hypothesis, mutations in genes which are also frequently mutated in other myeloid malignancies are also present at relatively high frequencies in AdvSM patients [10,11,13,90,91,92] (Figure 1). In this regard, it has been recently described that certain DNA methylation patterns may be relevant in the pathogenesis of systemic diseases associated with MC activation [93]. Moreover, a significant number of somatic mutations has been identified in a broad number of genes involved in epigenetic regulatory mechanisms, which have been associated, at least in part, with the pathogenesis, clinical behaviour and evolution of different myeloid neoplasms, including SM [94,95]. Thus, around 30–40% of AdvSM present with an associated myeloid haematological neoplasm already at diagnosis [69], suggesting a close relationship between both malignancies. In line with this, next generation sequencing (NGS) studies have confirmed the presence of recurrent mutations in genes involved in post-transcriptional mRNA processing, epigenetic modification of DNA and transcription and signal transduction factors, in both SM and other myeloid neoplasms [10,11,51,81,87]. Among others, mutations have been recurrently reported in AdvSM in the *ASXL1*, *CBL*, *DNMT3A*, *NRAS*, *RUNX1*, *SRSF2* and *TET2* genes in AdvSM [10,12,13,29,32,46,50,51,68,80,81,87,96,97]. In contrast, the presence of these additional mutations is a relatively infrequent finding in BMM and ISM patients [10,29,51,80,87].

### 4.1. Mutations Affecting Transcription Factors and Signalling Pathways

The correct function and development of the human organism strongly relies on the precise regulation and appropriate production of specific sets of proteins. Gene expression is largely regulated by transcription factors and the activation of processes involved in various intracellular signalling pathways. In this regard, alterations in genes involved in these processes, such as the *CBL*, *JAK2*, *K/NRAS* and/or *RUNX1* genes [104], have been associated with several haematological malignancies. To date, mutations in a total of 11 genes related to transcription factors and signalling pathways have been described in patients with different subtypes of SM; of note, while some of these genes have been sporadically reported to be mutated in SM (*EPHA7* [12,13], *FLT3* [29], *IKZF1* [13], *PIK3CD* [12,13], *ROS1* [12,13] and *TP53* [29]) (Tables S1 and S2), others (e.g., *CBL*, *JAK2*, *K/NRAS* and *RUNX1*) are recurrently found to be altered in SM, particularly among SM-AHN patients (Table 3).

The *CBL* (Casitas B-lineage lymphoma proto-oncogene) gene is located on chromosome 11 and encodes for a protein involved in the functional regulation (via competitive blockade) of tyrosine kinase (TK) receptors; in addition, the CBL product also acts in ubiquitination-mediated protein degradation in the proteasome [105,106]. Overall, mutations affecting the *CBL* gene in myeloid malignancies show a predominance of deletions involving the exon 8 of this gene [107] at frequencies that vary from 15% of patients diagnosed with juvenile myelomonocytic leukaemia, to 13% of CMML (mostly the *CBL* Y371 mutation) [108,109], 10% of AML and 8% of atypical chronic myeloid leukaemia cases [109,110,111]. Similarly, *CBL* mutations are found in a variable percentage of SM patients [10,11,29,46,68,80,81], where they are predominantly located at exon 8 (frequently also at codon Y371) (Table 2), their frequency ranging from <1% in Non-AdvSM patients to >10% of AdvSM cases [10,29,80,81,90], including >25% of SM-AHN patients in some cohorts [10,90] (average of 15%) (Table 3). In contrast to other myeloid neoplasms in which the impact of *CBL* mutations remains unclear [106,109,110,112], their presence in SM has been associated with poorer outcomes [90].

The *JAK2* (Janus Kinase 2) gene is located on human chromosome 9 and encodes a protein that acts as an intracellular (non-receptor) TK that is associated with various cell surface receptors for transducing activating signals through relevant pathways such as the mitogen-activated protein kinase (MAPK) and signal transducer and activator of transcription (STATs) pathways [98,113,114]. The most common *JAK2* activating mutation, the *JAK2* V617F mutation, has been reported in several diagnostic subtypes of MPN [115], which can explain its high incidence (about 11%) in SM-AHN patients [29,50,51,81,88] as compared to other diagnostic subtypes of SM [10,12,29,51,88] (Table 3 and Table S3). A recent study in SM-AHN patients showed that *KIT* D816V and *JAK2* V617F mutations probably arise in two independent clones in most patients, in which the presence of *JAK2* mutations appears to have a low prognostic impact [116].

The *KRAS* (Kirsten Rat Sarcoma Viral Oncogene Homolog) and *NRAS* (Neuroblastoma RAS Viral Oncogene Homolog) genes are both located on chromosome 12, and they encode proteins involved in signalling pathways associated with growth factor membrane receptors through their interaction with membrane GTPases. A large number of somatic mutations involving the *KRAS/NRAS* genes have been identified, mostly associated with solid tumours such as lung cancer, pancreatic cancer and colorectal cancer, among other prevalent tumours [117,118]; in some of these tumours such as metastatic colorectal cancer, *KRAS* and *NRAS* mutations have also been associated with a poorer prognosis [119]. In myeloid neoplasms, *NRAS* mutations have been associated with the development of AML (7–13%) secondary to different subtypes of MPN; however, it remains unclear whether these mutations directly promote progression to leukaemia [111]. With regards to SM, *KRAS* and/or *NRAS* mutations have been sporadically reported in ISM [12,97] and MCL cases [10,29], while they are more frequently found among SM-AHN patients, particularly in cases associated with poor-prognosis myeloid neoplasms (i.e., AML) [10,29,46,50,51,68,84] (Table 3 and Table S3); in this setting, some authors have suggested that these mutations might have an adverse prognostic impact [120].

*RUNX1* (Runt-Related Transcription Factor 1) is a gene located on human chromosome 21 that encodes a functional protein that acts as a transcription factor involved in the development of HSC [121]. The most frequent *RUNX1* mutations have been associated with progression from MPN to AML [122], which could explain the high frequency of these mutations (up to 37%) among patients with secondary AML [105,111]. In line with these findings, the presence of *RUNX1* mutations in patients with MDS is associated with resistance to specific chemotherapeutic drugs and shortened survival [123,124]. In SM, *RUNX1* mutations are preferentially located at exons 4 and 5 of the gene [10,11,12,13,29,32,68,97,125] (Table 2), with a frequency that ranges from <1% of Non-AdvSM patients to up to 18% of AdvSM cases, the highest frequency being detected in SM-AHN patients [10,13] (Table 3). From a prognostic point of view, *RUNX1*-mutated cases have been associated with an adverse outcome, both among Non-AdvSM and AdvSM patients [11,12,13,97,126].

### 4.2. Mutations in Genes Involved in Epigenetic Regulatory Mechanisms

Although the specific role of each individual epigenetic alteration detected in SM remains unknown [127,128,129,130], recurrent mutations in genes involved in epigenetic modifications of DNA (i.e., *ASXL1*, *CILK1*, *DNMT3A*, *EZH2*, *IDH1*, *IDH2*, *KAT6B*, *NPM1*, *SETBP1* and *TET2* genes) have been recurrently identified (Table 2, Tables S1 and S2); among these, mutations involving the *ASXL1*, *DNMT3A*, *EZH2* and *TET2* genes are the most commonly reported ones (Table 4).

The *ASXL1* (ASXL transcriptional regulator 1) gene encodes for a protein that interacts with the retinoic acid receptor involved in chromatin remodelling, although its precise function remains largely unknown [131]. The most frequent *ASXL1* mutations found in myeloid neoplasms are located at exon 12 [132], with an overall incidence that ranges from <7% of patients with essential thrombocytopenia (ET) or polycythaemia vera (PV), to almost 40% of primary myelofibrosis cases [133]. *ASXL1* is also the second most frequently mutated gene in MDS and CMML, and it is altered in up to 30% of AML patients [132,134]. Most reported *ASXL1* mutations in SM are also located at exon 12 [12,13,29,32,80,81] (Table 2) with a highly variable frequency that ranges from 1% of BMM cases to >20% of AdvSM patients, particularly of SM-AHN cases (Table 4). Similarly to other myeloid neoplasms [124,133,135], *ASXL1* mutations have been also (recurrently) associated with a worse prognosis in SM [11,29,68,80,81,85].

The *DNMT3A* (DNA Methyltransferase 3 Alpha) gene located on chromosome 2, encodes for an enzyme responsible for the methylation of CpG islands, which is critical in various physiological processes during embryogenesis and/or in the inactivation of the X chromosome [136]. The most frequently described mutation in the *DNMT3A* gene occurs at codon R882 [137], being present in 8–13% of MDS, 26% of AML secondary to MDS and 2% of CMML patients [137,138]. In general, the presence of *DNMT3A* mutations in patients with myeloid malignancies has been associated with a higher number of blasts in BM and greater leukocyte counts in blood [124,134] in the absence of a clear prognostic impact [134,137,138,139]. Although *DNMT3A* mutations have been described at relatively similarly low frequencies in Non-AdvSM and AdvSM (4% vs. 6%, respectively) (Table 4), their presence has been associated with a significantly poorer prognosis in some patient cohorts [12,80].

The *EZH2* (Enhancer of Zeste 2 polycomb repressive complex 2 subunit) gene encodes a protein of the PRC2 complex involved in proliferation, differentiation, ageing and maintenance of the chromatin structure through methylation, acting as both a tumour suppressor gene and an oncogene [105]. The *EZH2* gene is coded in chromosome 7, and its mutations have been described in both myeloid and lymphoid malignancies, as well as in solid tumours, where they have been recurrently associated with more advanced tumour stages and metastatic disease [140]. In myeloid neoplasms, *EZH2* mutations have been described in patients with PV (3%), myelofibrosis (13%), CMML (6%), AML (6%) and MDS (10%) [105,134,139,141,142]; in MDS they have been associated with a worse prognosis [124,142]. In SM, *EZH2* mutations have been reported almost exclusively within AdvSM patients [10,13,29,32,85], particularly among ASM and SM-AHN cases (Table 4).

The *TET2* (Ten–eleven translocation methylcytosine dioxygenase 2) gene is located on chromosome 4 and encodes for a protein that catalyses the conversion of 5-methylcytosine (5-mc) to 5-hydroxymethylcytosine (5-hmc) in the DNA [143]. It is believed that 5-hmc may initiate DNA demethylation by preventing binding to the CpG islands of DNA methyltransferases characteristic of these sequences [144]. To date, *TET2* mutations have been described in every exon of the gene, and sometimes mutations involving both alleles coexist in the same cell [13,145]. *TET2* mutations are considered to be early events in the development of haematological malignancies such as MPN, MDS, CMML and different subtypes of leukaemia and lymphoma, as well as in SM [145]. Overall, *TET2* mutations have been described in about 14% of MPN, 23% of MDS (in which they usually occur together with mutations in *SF3B1*, *U2AF1*, *ASXL1*, *SRSF2* and/or *DNMT3A* and also a normal karyotype [124]) and 30% of CMML patients (often associated with mutations in the *SRSF2* and *U2AF1* genes) [10,91,124,134,146]. In SM, *TET2* is the most frequently mutated gene other than *KIT*. In these later patients, *TET2* mutations have been reported along the entire gene sequence but more frequently at exons 3, 9 and 11 (Table 2). As found also in MDS, the coexistence of *TET2* and *SRSF2* gene mutations has also been reported in SM [10,85]. Of note, in vitro studies suggest that in a significant proportion of patients with SM-AHN, *TET2* mutations may precede the *KIT* D816V mutation [85], similarly to what would also occur with *ASXL1* and *SRSF2* mutations. However, despite *TET2* mutations being significantly more frequently detected in AdvSM vs. Non-AdvSM patients (39% vs. 3% of the cases, respectively) [10,12,13,32,50,51,68,80,81,85,88,90] (Table 4), and their being associated with the presence of C-findings [51], they do not seem to have any prognostic impact in SM [10,11,12,13,29,68,80,81,97,139].

### 4.3. Mutations in Genes Involved in Alternative mRNA Splicing

The presence of mutations in genes associated with the spliceosome, responsible for alternative RNA processing, has been linked to different diagnostic subtypes of haematopoietic malignancies (e.g., MDS) and some solid tumours (e.g., ocular uveal melanoma or pulmonary fibrosis) [86,147]. These include mutations in the *SF3B1*, *SRSF2* and *U2AF1* genes, from which mutations in the former two genes have been described in SM at relatively high frequencies in SM (Table 5) and/or (i.e., *SRSF2*) in association with poorer outcomes [11,96].

The *SRSF2* (serine and arginine rich splicing factor 2) gene encodes for a protein that is critical for alternative mRNA processing at the post-transcriptional level [148], which also acts as an important regulator of DNA stability, being a key player in the DNA acetylation/phosphorylation network [149]. The most frequent somatic mutations of *SRSF2* found in SM patients are located at codon P95 [10,12,13,32,87] (Table 2). Among patients with other myeloid haematological neoplasms, *SRSF2* mutations are particularly frequent (28–30%) among CMML cases [150] and, to a less extent, MDS (11%) and AML (6%) patients [124,134,150]. Recent studies in SM patients show the presence of *SRSF2* mutations in a variable percentage of cases ranging from <1% of Non-AdvSM cases to around one third of AdvSM patients (Table 5), being one of the most frequently mutated genes in SM, particularly in SM-AHN cases [10,11,12,13,29,32,46,50,68,80,87,97]. In contrast to other haematological neoplasms [134,151,152,153], the presence of *SRSF2* mutations has been consistently associated with an adverse prognosis in patients with SM [13,97], particularly among AdvSM cases [11,46,68].

The *SF3B1* (splicing factor 3b subunit 1) gene is located in chromosome 2, and it encodes for the largest subunit of the SF3B complex, a core component of the U2 small nuclear ribonucleoprotein of the U2-dependent spliceosome [154]. *SF3B1* is the most commonly mutated splicing factor gene in MDS patients [155], in whom it is associated with a more favourable outcome [156]. In contrast to *SRSF2*, *SF3B1* mutations have been less frequently described in SM [12,13,29,32,83,86], with only the K666 codon found to be mutated in more than two patient series. Actually, *SF3B1* mutations are detected in <7% of AdvSM patients (most frequently in SM-MDS cases [13,68,85]) (Table S3), while they are rarely found in Non-AdvSM patients [10,12,29,68] (Table 5). Likewise, *U2AF1* mutations are also relatively rare in SM, with a higher incidence in AdvSM [10,29,50] vs. Non-AdvSM cases (6% vs. 1%, respectively) (Table S2); these mutations are mostly located at codons S34 [29,157,158] and Q157 [29] of the *U2AF1* gene (Table S1).

## 5. Prognostic Impact of Acquired Gene Mutations in Systemic Mastocytosis

Acquisition of the *KIT* D816V mutation in HSC during haematopoiesis leads to multilineage involvement by the *KIT* mutation [77], which is associated with a poor prognosis of Non-AdvSM cases due to an increased risk of progression to AdvSM [5,77]. Despite the relatively early onset of the *KIT* D816V mutation throughout life, in at least a fraction of (i.e., multilineal) SM patients [159], the most common clinical manifestations of the disease (e.g., urticaria pigmentosa and/or anaphylaxis) usually emerge at the third or fourth decades of life in the majority of SM cases [9,159,160]. Thus, from the constitutive activation of KIT in HSC until the development of an advanced form of SM, progressive expansion and accumulation of mutated cells is required to occur, probably in association with the acquisition of secondary genetic lesions, an increased capacity to maintain them (e.g., activation/repression of anti-/pro-apoptotic mechanisms) [79] and/or the cooperation with a specific genetic background [161]. Studies performed in murine models and in patients with SM have shown that the coexistence of the *KIT* D816V mutation and mutation(s) in genes other than *KIT* are probably necessary for the progression and transformation from pauci-symptomatic Non-AdvSM to advanced forms of the disease [10,51,159,162]. However, neither a specific mutation (or mutation profile) nor a specific genetic background shared by all AdvSM patients have been identified so far [10,11,13,51,80,81,85,87,88,137]. Instead, the number of mutated genes (other than *KIT*) significantly increases from ISM to ASM [13,46] and other subtypes of AdvSM [10,29,163]. Of note, the acquisition of these additional (somatic) mutations in ISM patients who present with the multilineage *KIT* D816V mutation is usually associated with progression of the disease to, e.g., SSM and/or ASM [12,32]. In fact, demonstration of multilineage involvement of haematopoiesis by the *KIT* D816V mutation has been shown to be an independent prognostic factor for predicting progression of ISM [5]. In line with these findings, most AdvSM cases carry the multilineage *KIT* D816V mutation associated with involvement of CD34^+^ HSC, except for a minor fraction of SM-AHN patients that have the *KIT* D816V mutation restricted to the MC compartment in BM. Interestingly, in these latter cases, the SM and AHN components of the disease appear to derive from independent clones that coexist in the same individual [36]. Despite the prognostic relevance of the multilineage *KIT* mutation, access to BM cell purification techniques required to investigate the presence of the *KIT* mutation in different myeloid and lymphoid compartments of BM cells is still restricted to a limited number of diagnostic laboratories, which has hampered the use of the multilineage *KIT* mutation as a predictor for the progression of ISM to SSM and AdvSM in routine diagnostics [7,32,120]. In line with this, the use of high sensitivity (quantitative) methods for the identification of the *KIT* D816V mutation [164,165,166] has proven in recent years to be of great utility to identify ISM patients that present the multilineage *KIT* as those that display a high *KIT* D816V variant allele frequency (VAF) in unfractionated BM (i.e., VAF ≥ 1–2%) [12,167] and/or blood (VAF ≥ 6%). [78] In fact, these later ISM cases also carry a significantly higher probability of undergoing disease progression associated with a significantly shortened life expectancy [167]. These findings support the use of a high *KIT* D816V VAF (as assessed by allele-specific qPCR) as a surrogate marker of multilineage involvement of haematopoiesis by the *KIT* D816V mutation [168].

In parallel, several studies on the general adult population have shown that the presence of mutations in genes that are considered to be initiators (or “drivers”) of clonal expansions of HSC [169,170], while exceptional among individuals <40 years of age [170], progressively increases from the fifth decade of life onwards [171,172], being recognized as age-related clonal haematopoiesis (ARCH). Of note, ARCH is characterized by the presence of somatic mutations in genes that are also frequently mutated in SM (e.g., *ASXL1*, *DNMT3A*, *EZH2*, *RUNX1*, *SF3B1*, *SRSF2* and *TET2*) and other myeloid neoplasms [92,173,174]. Some of these ARCH-related genetic mutations that are frequently reported in AdvSM cases have been confirmed to be directly associated with the development of haematopoietic neoplasms and are considered clonal haematopoiesis of oncogenic potential (CHOP) mutations [175] (e.g., *SRSF2* [11], *ASXL1* [11], *DNMT3A* [80], *RUNX1* [11], *EZH2* [13], *CBL* [90]). These findings may contribute to explain the higher prevalence of myeloid neoplasms among older individuals and would be in agreement with the observation that age ≥60 years at diagnosis of SM predicts an increased risk of (primary and secondary) AdvSM [7,32,120]. Therefore, acquisition of ARCH-related gene mutations is currently considered to be closely associated with (a higher risk for) more advanced forms of SM. Among AdvSM patients, mutations in most of these genes (e.g., *ASXL1*, *CBL*, *JAK2*, *KRAS*, *NRAS*, *RUNX1*, *SRSF2* and *TET2*) have been reported to be more frequently associated with SM-AHN than ASM, with only a few exceptions that involve genes that show similarly mutated frequencies in both subtypes of SM (i.e., *DNMT3A*, *EZH2* and *SF3B1*) (Table 4 and Table 5). Moreover, for most of these mutated genes (e.g., *ASLXL1*, *DNMT3A*, *EZH2*, *IKZF1*, *RUNX1*, *SF3B1*, *SRSF2* and *TET2*) [12], a high VAF is usually detected in the BM of AdvSM patients, which might also reflect the presence of multilineage involvement of haematopoiesis by these mutations, similarly to what has been described above for *KIT* D816V [9,13,74]. In these multi-mutated SM patients, the exact sequence of acquisition of genetic mutations remains unclear; thus, in some patients, the *KIT* D816V appears to be the first acquired mutation [13], while another subgroup of SM cases carries the *KIT* D816V mutation and mutations in genes other than *KIT* in different cell clones [13,36,85], and in a third subgroup of SM patients, the *KIT* mutation appears to be a secondary event. Of note, the two later subgroups of patients are usually diagnosed with SM associated with another myeloid neoplasm (i.e., SM-AHN) [13,36,85].

Altogether, these observations suggest that in patients with Non-AdvSM, the disease is mostly driven by the *KIT* D816V mutation, while the occurrence of additional mutations in other genes would be required (prior to or after the *KIT* mutation) for the development of AdvSM. In order to elucidate whether any of these mutated genes confers an adverse prognosis, multiple studies have been conducted in SM [11,12,13,29,32,68,80,90,120,160,176], from which a few include medium to large patient cohorts (*n* ≥ 100) [12,29,32,68,97]. Of note, the number of genes screened in these studies is highly variable, ranging from 9 genes [80] to 410 genes [13], with a few patients being investigated by whole-genome [13] or whole-exome [42,177,178,179] sequencing. Overall, these studies found a total of 30 different genes to be mutated in SM (Table 2 and Table S1), from which 11 (i.e., *ASXL1*, *CBL*, *DNMT3A*, *EZH2*, *JAK2*, *KRAS*, *NRAS*, *RUNX1*, *SF3B1*, *SRSF2*, *TET2*) are recurrently mutated genes in several SM cohorts (Table 3, Table 4 and Table 5). From these later 11 mutated genes, a few have (independent) prognostic implications as regards disease progression and/or overall patient survival (i.e., *SRSF2* [11,32], *ASXL1* [11,32], *DNMT3A* [12], *RUNX1* [11,32], *EZH2* [13], *CBL* [90] and *NRAS* [120]), particularly when mutation/s are present at high VAF [12]. Because of this, the presence of mutations in limited sets of genes has been included in several recently proposed risk stratification models for both AdvSM (i.e., *SRSF2/ASXL1/RUNX1* [11], *SRSF2/ASXL1/RUNX1/EZH2* [13], *ASXL1/RUNX1/NRAS* [120]) and Non-AdvSM (e.g., *ASXL1/RUNX1/DNMT3A* [12]). The recent development of SM-induced pluripotent stem cells (iPSCs) positive for *KIT* D816V and other concurrent mutations [180], which accurately reflect the genetic background of SM patients’ multi-mutated pathological cells, may become a powerful tool to dissect the impact of these mutations on the aetiopathogenic mechanisms involved in disease progression [181]. Therefore, molecular characterization of the genetic background of AdvSM patients, including NGS as described above, VAF assessment of somatic mutations [125,182] and drug screening in patient-derived iPSCs [180,183], may lead to improved molecularly targeted treatment options in a context of personalized precision medicine.

## 6. Conclusions

At present it is well-established that SM is a clonal HSC disease characterized by the expansion and accumulation of neoplastic MCs, where the presence of activating *KIT* mutations (most commonly *KIT* D816V) is a hallmark of the disease, being present in most (>90%) adult patients, at similar frequencies in Non-AdvSM and AdvSM. Despite the *KIT* D816V mutation being currently considered the pathogenic driver of SM, it cannot explain by itself the heterogeneous clinical behaviour of this disease. In this regard, the presence of multilineage involvement of haematopoiesis by the *KIT* D816V mutation, particularly in the context of a multi-mutated disease in which additional myeloid-neoplasm-associated genes other than *KIT* are also mutated, emerges as the altered genetic background that might contribute to explain malignant transformation of SM. Because of this, assessment of multilineage involvement of haematopoiesis by the *KIT* D816V mutation should be performed in newly diagnosed SM patients to identify those cases at high risk of progression to AdvSM. In addition, identification of other pathogenic mutations in genes with known prognostic impacts in SM (i.e., *SRSF2*, *ASXL1*, *DNMT3A*, *RUNX1*, *EZH2*, *CBL* and *NRAS*) should also be performed in SM patients with multilineage involvement of haematopoiesis by *KIT* D816V for further identification of patients at higher risk of death who may benefit from a closer follow-up and eventually, also, early cytoreductive treatment. Just as nowadays the measurement of allele burden of the *KIT* D816V mutation has become an important predictor of treatment response assessment [182] and survival [125], further analysis of the VAF for these later genes might provide a more reliable marker for assessing tumour burden as compared to other clinical and/or laboratory parameters, which can be also altered by medication or intercurrent processes (e.g., infectious and/or allergic diseases) [184,185,186]. Importantly, all above molecular markers should be used in combination with other disease features for accurate risk stratification of SM patients [12,32,97,120].

## Figures and Tables

**Table 1:** *KIT* mutations other than D816V described in adult patients with systemic mastocytosis (SM).

Domain	Exon	Mutation	Subtype SM
Extracellular: Ligand (SCF) binding domain	2	R49C	SM-u [29]
	5	Y269C	SM-AHN [40]
Extracellular: Dimerization domain	7	V399I	SM-u [29]
	8	D419del	ISM [47]
	9	S451C	SM-u [61]
		S476I	MCL [48]
		S501_A502dup	ASM [50] MCL [49]
		A502_Y503dup	SM-u [29] MCL [51,52]
		Y503_F504InsAY	ASM [40]
		F504_N505delIns5	SM-AHN [51]
		K509R	SM-u [29]
		K509I	ISM [62] ASM [53] MCL [62] WDSM [63]
Transmembrane domain	10	F522C	WDSM [32,54,55] MCL [46]
Juxta-membrane domain	11	V559I	ASM [56]
		V560G	SM-u [40] ISM [57] MCL [58]
TK1 domain	13	K642E	ASM [29,40]
		V654A	MCL [59]
TK2 domain	17	A814S	SM-AHN [26]
		A814T	SM-AHN [27]
		I815-V816Ins	ISM [9]
		D816H	AdvSM [32,33] SM-AHN [26,34,35,36] MCL [46,67,68]
		D816Y	ISM [37] AdvSM [32,33] SM-AHN [26,27,36,38,39] MCL [9,46]
		D816I	SM-AHN [40]
		D816A	SM-AHN [41,45] ASM [42]
		D816G	MCL [43]
		D816T	SM-u [44]
		I817V	WDSM [9]
		D820G	ASM [28]
		N822K	SM-u [30] SM-AHN [31]
	18	V852G	SM-u [29]

Abbreviations: AdvSM: advanced systemic mastocytosis (SM); ASM: aggressive SM; ISM: indolent SM; MCL: mast cell leukaemia; SM-AHN: SM with an associated haematological neoplasm; SM-u: SM unclassified; WDSM: well-differentiated SM.

**Table 2:** Mutations in genes other than *KIT* reported in systemic mastocytosis.

Gene	Exon	Gene Mutations				
*ASXL1*	6	S135C [12]				
	8	G219V [12]				
	12	Y591* [80]	I641fs [13]	P698Afs* [32]	I919Yfs* [29]	H1008Tfs* [29]
		E602* [29]	G642fs [12]	R786Efs* [29]	P920Tfs* [80]	G1026Dfs* [29]
		A611T [29]	G643Wfs* [13]	D820Mfs* [29]	R965_G966del [12]	I1220F [80]
		I617Pfs* [32]	G646Wfs* [29,81]	T844fs [12]	Y974* [32]	G1397S [29]
		H630fs [12]/Gfs* [29]	G646Afs* [29]	S846Vfs* [81]	I980Kfs* [32]	A1521S [12]
		E635Rfs* [13,29,32]	R693* [13]	P849Lfs* [29]	E997* [29]	*1542fs [13]
*CBL*	8	Q367dup [80]	Y371C [29]/H [29]/S [29]		M374K [29]	L380P [10,29,82]
		C384R [12]/Y [29]	M400K [10,32]	C404Y [10,29]	W408C [10,32]	
	9	G413D [29]	R420Q [10,29]	I423N [29]		
	11	R550W [12]				
*DNMT3A*	3	E30A [80]				
	4	N90S [12]				
	8	R320* [12,29]				
	10	A380V [12]	K420* [12]			
	15	W581C [12]	L594Cfs* [29]	R598* [29]	D600Afs* [29]	
	16	S638C [12]				
	17	S663L [12]				
	18	S714F [12]	R720L [12]			
	19	E733G [29]	F755S [12]	R771G [29]/Q [80]		
	23	N879D [13]	R882C [12,13]/H [12,13,80]			
*EZH2*	3	L50Wfs* [13]				
	5	I146T [13]				
	7	S220F [32]				
	8	R288* [32]				
	14	Q545* [13]				
	15	R583Q [13]	N608K [13]			
	17	F672L [29]				
	18	R690C [29]	H694R [40]			
*JAK2*	14	V617F [12,29,32]	S605Y [12]			
*KRAS*	2	V14I [83]				
	3	Q70H [12]				
	5	I187N [12]				
*NRAS*	2	G12S [29]	G12D [29,84]	G13D [84]		
	3	Q61L [29]				
*RUNX1*	4	L56S [29]	P86fs [13]		E88Rfs* [13]	S94I [29]
		D96Gfs [85]	R107C [13]		N109T [13]/del [29]	F116L [12]
	5	S141L [29]	A142T [13]		N146K [12,13]	R162K [13,29]
	7	V238Gfs* [13]				
*SF3B1*	5	Y141C [12]				
	14	R625C [29]	W658C [29]	T663I [29]	K666N [29]/T [13,32]	
	15	K700E [13,29,83]	A711D [86]			
*SRSF2*	1	V18L [10]	P95 A [13]/H [10,32,85]/L [10]/R [12,13]/T [87]			
*TET2*	3	L34F [29]	Q321* [83]	P562Tfs* [29]	Q729* [32]	Q933* [51]
		H192Y [13]	E368* [13]	N595Ifs* [51]	Q731* [51]	Q939* [29]
		V218Wfs* [32]	Q373Rfs* [51]	P612fs [12]	Q734* [51]	K948Nfs* [83]
		Y234* [51]	P409Lfs* [80]	L615Sfs* [29]	Q752_fs* [29]	W954* [29]
		R248Q [29]	G429R [29]	Y620fs [12]	L757Tfs* [29]	Q958Tfs* [29]
		S254Rfs* [51]	L431* [51]	Y634* [29]	L806Rfs* [29]	Q963* [29]
		E259Gfs* [29]	E452Rfs* [29,88]	Q652* [29]	Q810* [29,88]	S972Ffs* [29]
		N275Ifs* [12,13]	D527Gfs* [29]	Q652Sfs* [80]	N837Yfs* [32]	C1016Wfs* [29,88]
		Q278* [29]	Q530* [29]	Q659Rfs* [29]	L840* [51]	Q1020* [80]
		T279fs [12]	E537Sfs* [29]	Q684Nfs* [29]	T849Hfs* [89]	I1024Qfs* [51]
		N281* [29]	R550* [29]	Q705Sfs* [29]	Y867H [29]	P1061Qfs* [51]
		R282G [29]	H558Lfs* [51]	A727S [12]	V872Cfs* [29]	Q1084P [29]
	4	D1143Mfs* [32,51]				
	5	Q1170* [88]				
	6	H1219D [32]	Y1245Lfs [85]	S1246* [51]	Y1255fs [12]	
	8	Y1337* [80]	A1341E [85]			
	9	Y1351* [51]	R1359 C [88]/H [29]	S1369L [29]	H1380Y [29]	D1384V [29]
		Q1389* [51]	T1393I [29]			
	10	R1465* [29]	R1467G_fs* [29]	K1493fs [12]		
	11	L1515Ffs* [51]	M1615* [89]	V1718L [29]	L1819* [29]	N1890S [12]
		L1531A_fs* [29,88]	Q1652Hfs* [29]	P1723S [29]	I1873T [80]	R1891G [29]
		K1533* [29]	Y1679L_fs* [29]	D1750Efs* [51]	E1879* [29]	F1901Lfs* [51]
		E1555R_fs* [29]	Q1680* [29]	N1765* [29]	H1881L [29]/R [29,51,88]	Y1902C [29]
		Y1598Sfs* [29]	S1688_fs* [29,51,88]	M1800Dfs* [51]	T1884A [29]	H1904R [80]
		S1611Y [12]	M1701I [29]	H1817Pfs* [29]	L1886S [29]	H1912Y [13]

*: Stop codon resulting in an incomplete protein.

**Table 3:** Frequency of mutations in genes affecting transcription factors and signalling pathways found to be recurrently altered in systemic mastocytosis.

Gene	SM Diagnostic Subgroup	Mutated Cases/Total Cases (%)	Overall Frequency	WHO Subtype	Mutated Cases/ Total Cases (%)		Overall Frequency
*CBL*	Non-AdvSM	1/12 (0) [10] 1/216 (0.5) [12] 0/6 (0) [13] 0/44 (0) [29] 0/1 (0) [50] 1/29 (3.4) [51] 0/26 (0) [68] 0/15 (0) [80] 0/6 (0) [85]	1%	BMM	0/65 (0) [12]		0%
				ISM	1/10 (10) [10] 0/3 (0) [13] 0/1 (0) [50] 0/26 (0) [68] 0/4 (0) [85]	1/144 (1) [12] 0/44 (0) [29] 1/28 (4) [51] 0/15 (0) [80]	1%
				SSM	0/2 (0) [10] 0/3 (0) [13] 0/2 (0) [85]	0/7 (0) [12] 0/1 (0) [51]	0%
	AdvSM	7/27 (26) [10] 1/13 (8) [12] 0/14 (0) [13] 16/106 (15) [29] 25/272 (9) [32] 1/25 (4) [50] 0/35 (0) [51] 10/83 (12) [68] 1/10 (10) [80] 3/26 (12) [81] 2/13 (15) [85] 4/19 (21) [90]	11%	ASM	0/1 (0) [10] 0/9 (0) [13] 0/2 (0) [50] 0/3 (0) [68] 0/1 (0) [85]	0/9 (0) [12] 1/25 (4) [29] 0/9 (0) [51] 0/2 (0) [80] 0/6 (0) [90]	2%
				SM-AHN	6/23 (26) [10] 0/5 (0) [13] 1/21 (5) [50] 10/72 (14) [68] 2/12 (17) [85]	1/4 (25) [12] 15/80 (19) [29] 0/23 (0) [51] 3/26 (12) [81] 4/13 (31) [90]	15%
				MCL	2/7 (29) [10] 0/2 (0) [50] 0/8 (0) [68]	0/1 (0) [29] 0/3 (0) [51]	10%
*JAK2*	Non-AdvSM	0/12 (0) [10] 2/97 (2) [12] 0/6 (0) [13] 0/44 (0) [29] 0/1 (0) [50] 0/29 (0) [51] 2/26 (8) [68] 0/6 (0) [85] 0/13 (0) [88]	2%	BMM	1/23 (4) [12]		4%
				ISM	0/10 (0) [10] 0/3 (0) [13] 0/1 (0) [50] 2/26 (8) [68] 0/13 (0) [88]	1/70 (1) [12] 0/44 (0) [29] 0/28 (0) [51] 0/4 (0) [85]	2%
				SSM	0/2 (0) [10] 0/3 (0) [13] 0/2 (0) [85]	0/4 (0) [12] 0/1 (0) [51]	0%
	AdvSM	2/27 (7) [10] 0/14 (0) [13] 9/106 (9) [29] 25/213 (12) [32] 3/25 (12) [50] 3/35 (9) [51] 12/83 (15) [68] 3/47 (6) [81] 1/13 (8) [85] 2/29 (7) [88]	10%	ASM	0/1 (0) [10] 0/9 (0) [13] 0/2 (0) [50] 0/3 (0) [68] 0/5 (0) [88]	0/7 (0) [12] 0/25 (0) [29] 0/9 (0) [51] 0/1 (0) [85]	0%
				SM-AHN	2/23 (9) [10] 0/5 (0) [13] 3/21 (14) [50] 12/72 (17) [68] 1/12 (8) [85]	0/3 (0) [12] 9/80 (11) [29] 3/23 (13) [51] 3/47 (6) [81] 2/23 (9) [88]	11%
				MCL	0/3 (0) [10] 0/1 (0) [50] 0/8 (0) [68]	0/1 (0) [29] 0/3 (0) [51] 0/1 (0) [88]	0%
*KRAS*	Non-AdvSM	0/12 (0) [10] 2/97 (2) [12] 0/6 (0) [13] 0/29 (0) [51] 0/36 (0) [84]	1%	BMM	0/23 (0) [12]		0%
				ISM	0/10 (0) [10] 0/3 (0) [13] 0/27 (0) [84]	2/70 (3) [12] 0/28 (0) [51]	1%
				SSM	0/2 (0) [10] 0/3 (0) [13] 0/9 (0) [84]	0/4 (0) [12] 0/1 (0) [51]	0%
	AdvSM	4/27 (15) [10] 0/10 (0) [12] 0/14 (0) [13] 0/35 (0) [51] 2/16 (13) [68]	6%	ASM	0/1 (0) [10] 0/9 (0) [13]	0/7 (0) [12] 0/9 (0) [51]	0%
				SM-AHN	4/23 (17) [10] 0/5 (0) [13] 2/16 (13) [68]	0/3 (0) [12] 0/23 (0) [51]	9%
				MCL	0/3 (0) [10]	0/2 (0) [51]	0%
*NRAS*	Non-AdvSM	0/12 (0) [10] 0/6 (0) [13] 0/44 (0) [29] 0/1 (0) [50] 0/23 (0) [51] 0/36 (0) [84] 1/298 (0.3) [97]	0.2%	BMM			
				ISM	0/10 (0) [10] 0/44 (0) [29] 0/22 (0) [51]	0/3 (0) [13] 0/1 (0) [50] 0/27 (0) [84]	0%
				SSM	0/2 (0) [10] 0/1 (0) [51]	0/3 (0) [13] 0/9 (0) [84]	0%
	AdvSM	2/27 (7) [10] 0/14 (0) [13] 3/105 (3) [29] 1/25 (4) [50] 1/25 (4) [51] 2/16 (13) [68] 3/173 (2) [97]	3%	ASM	0/1 (0) [10] 0/25 (0) [29] 0/7 (0) [51]	0/9 (0) [13] 0/2 (0) [50]	0%
				SM-AHN	2/23 (9) [10] 3/80 (4) [29] 1/16 (6) [51]	0/5 (0) [13] 1/21 (5) [50] 2/16 (13) [68]	6%
				MCL	0/3 (0) [10] 0/2 (0) [50]	1/1 (100) [29] 0/2 (0) [51]	13%
*RUNX1*	Non-AdvSM	0/12 (0) [10] 1/309 (0.3) [12] 2/10 (20) [13] 0/44 (0) [29] 0/26 (0) [68] 0/6 (0) [85] 1/530 (0.2) [97]	0.4%	BMM	1/90 (1) [12]		1%
				ISM	0/10 (0) [10] 0/3 (0) [13] 0/26 (0) [68]	0/211 (0) [12] 0/44 (0) [29] 0/4 (0) [85]	0%
				SSM	0/2 (0) [10] 2/7 (29) [13]	0/8 (0) [12] 0/2 (0) [85]	11%
	AdvSM	9/27 (33) [10] 0/13 (0) [12] 7/24 (29) [13] 5/106 (5) [29] 66/329 (20) [32] 15/83 (18) [68] 1/13 (8) [85] 38/210 (18) [97]	18%	ASM	1/1 (100) [10] 2/11 (18) [13] 1/3 (33) [68]	0/9 (0) [12] 0/25 (0) [29] 0/1 (0) [85]	8%
				SM-AHN	8/23 (35) [10] 5/13 (39) [13] 14/72 (19) [68]	0/4 (0) [12] 5/80 (6) [29] 1/12 (8) [85]	16%
				MCL	0/3 (0) [10] 0/8 (0) [68]	0/1 (0) [29]	0%

Overall frequencies represent the weighted average of the percentage of patients with at least one mutation in that gene. out of the total number of patients studied within the different cohorts, for each SM subgroup. Abbreviations: AdvSM: advanced systemic mastocytosis (SM); ASM: aggressive SM; BMM: bone marrow mastocytosis; ISM: indolent SM; MCL: mast cell leukaemia; Non-AdvSM: non-advanced SM; SM-AHN: SM with an associated haematological neoplasm; SSM: smouldering SM.

**Table 4:** Frequency of mutations in genes involved in epigenetic regulatory mechanisms found to be recurrently altered in systemic mastocytosis.

Gene	SM Diagnostic Subgroup	Mutated Cases/Total Cases (%)	Overall Frequency	WHO Subtype	Mutated Cases/ Total Cases (%)		Overall Frequency
*ASXL1*	Non-AdvSM	0/12 (0) [10] 6/309 (2) [12] 0/10 (0) [13] 0/44 (0) [29] 0/1 (0) [50] 0/26 (0) [68] 1/15 (7) [80] 0/6 (0) [85] 6/530 (1) [97]	1%	BMM	1/90 (1) [12]		1%
				ISM	0/10 (0) [10] 0/3 (0) [13] 0/1 (0) [50] 1/15 (7) [80] 6/530 (1) [97]	4/211 (2) [12] 0/44 (0) [29] 0/26 (0) [68] 0/4 (0) [85]	1%
				SSM	0/2 (0) [10] 0/7 (0) [13]	1/8 (13) [12] 0/2 (0) [85]	5%
	AdvSM	8/27 (30) [10] 2/13 (15) [12] 2/24 (8) [13] 25/106 (24) [29] 66/229 (29) [32] 12/25 (48) [50] 21/83 (25) [68] 2/10 (20) [80] 6/43 (14) [81] 5/13 (39) [85] 5/19 (26) [90] 35/210 (17) [97]	24%	ASM	0/1 (0) [10] 1/11 (9) [13] 0/2 (0) [50] 0/2 (0) [80] 1/6 (17) [90]	1/9 (11) [12] 4/25 (16) [29] 0/3 (0) [68] 0/1 (0) [85]	9%
				SM-AHN	8/23 (35) [10] 1/13 (8) [13] 4/21 (19) [50] 6/43 (14) [81] 4/13 (31) [90]	1/4 (25) [12] 21/80 (26) [29] 21/72 (29) [68] 5/12 (42) [85]	25%
				MCL	0/3 (0) [10] 0/2 (0) [50]	0/1 (0) [29] 0/8 (0) [68]	0%
*DNMT3A*	Non-AdvSM	14/309 (5) [12] 0/10 (0) [13] 2/44 (5) [29] 0/26 (0) [68] 2/15 (13) [80] 20/530 (4) [97]	4%	BMM	2/90 (2) [12]		2%
				ISM	10/211 (0.5) [12] 2/44 (5) [29] 2/15 (13) [80]	0/3 (0) [13] 0/26 (0) [68]	5%
				SSM	2/8 (25) [12]	0/7 (0) [13]	13%
	AdvSM	4/13 (31) [12] 3/24 (13) [13] 7/106 (7) [29] 1/83 (1) [68] 1/10 (10) [80] 2/19 (11) [90] 9/210 (4) [97]	6%	ASM	3/9 (33) [12] 0/25 (0) [29] 0/2 (0) [80]	3/11 (27) [13] 0/3 (0) [68] 0/6 (0) [90]	11%
				SM-AHN	1/4 (25) [12] 7/80 (9) [29] 1/8 (13) [80]	0/13 (0) [13] 1/72 (1) [68] 2/13 (15) [90]	6%
				MCL	0/1 (0) [29]	0/8 (0) [68]	0%
*EZH2*	Non-AdvSM	0/12 (0) [10] 0/309 (0) [12] 1/10 (10) [13] 0/44 (0) [29] 0/26 (0) [68] 0/15 (0) [80] 0/6 (0) [85]	0.2%	BMM	0/90 (0) [12]		0%
				ISM	0/10 (0) [10] 0/3 (0) [13] 0/26 (0) [68] 0/4 (0) [85]	0/211 (0) [12] 0/44 (0) [29] 0/15 (0) [80]	0%
				SSM	0/2 (0) [10] 1/7 (14) [13]	0/8 (0) [12] 0/2 (0) [85]	5%
	AdvSM	2/27 (7) [10] 0/13 (0) [12] 4/24 (17) [13] 3/106 (3) [29] 17/305 (6) [32] 2/83 (2) [68] 0/10 (0) [80] 2/13 (15) [85]	5%	ASM	0/1 (0) [10] 2/11 (18) [13] 0/3 (0) [68] 0/1 (0) [85]	0/9 (0) [12] 1/25 (4) [29] 0/2 (0) [80]	6%
				SM-AHN	2/23 (9) [10] 2/13 (15) [13] 2/72 (3) [68] 2/12 (17) [85]	0/4 (0) [12] 2/80 (3) [29] 0/8 (0) [80]	5%
				MCL	0/3 (0) [10] 0/8 (0) [68]	0/1 (0) [29]	0%
*TET2*	Non-AdvSM	0/12 (0) [10] 7/309 (2) [12] 0/10 (0) [13] 0/1 (0) [50] 2/29 (7) [51] 1/26 (4) [68] 1/15 (7) [80] 0/6 (0) [85] 2/13 (15) [88]	3%	BMM	2/90 (2) [12]		2%
				ISM	0/10 (0) [10] 0/3 (0) [13] 1/28 (4) [51] 1/15 (7) [80] 2/13 (15) [88]	5/211 (2) [12] 0/1 (0) [50] 1/26 (4) [68] 0/4 (0) [85]	3%
				SSM	0/2 (0) [10] 0/7 (0) [13] 0/2 (0) [85]	0/8 (0) [12] 1/1 (100) [51]	5%
	AdvSM	15/27 (56) [10] 2/13 (15) [12] 3/24 (13) [13] 137/329 (42) [32] 12/25 (48) [50] 12/35 (34) [51] 33/83 (40) [68] 5/10 (50) [80] 12/32 (38) [81] 6/13 (46) [85] 10/29 (35) [88] 5/19 (26) [90]	39%	ASM	0/1 (0) [10] 0/11 (0) [13] 3/9 (33) [51] 0/2 (0) [80] 2/5 (40) [88]	1/9 (11) [12] 2/2 (100) [50] 0/3 (0) [68] 0/1 (0) [85] 1/6 (17) [90]	21%
				SM-AHN	15/23 (65) [10] 3/13 (23) [13] 9/23 (39) [51] 5/8 (63) [80] 6/12 (50) [85] 4/13 (31) [90]	1/4 (25) [12] 9/21 (43) [50] 33/72 (46) [68] 12/32 (38) [81] 8/23 (35) [88]	43%
				MCL	0/3 (0) [10] 0/3 (0) [51] 0/1 (0) [88]	1/2 (50) [50] 0/8 (0) [68]	6%

Overall frequencies represent the weighted average of the percentage of patients with at least one mutation in that gene out of the total number of patients studied in the different cohorts for each SM subgroup. Abbreviations: AdvSM: advanced systemic mastocytosis (SM); ASM: aggressive SM; BMM: bone marrow mastocytosis; ISM: indolent SM; MCL: mast cell leukaemia; Non-AdvSM: non-advanced SM; SM-AHN: SM with an associated haematological neoplasm; SSM: smouldering SM.

**Table 5:** Frequency of mutations in genes involved in alternative mRNA splicing recurrently found in systemic mastocytosis.

Gene	SM Prognostic Subgroup	Mutated Cases/ Total Cases (%)	Overall Frequency	WHO Subtype	Mutated Cases/ Total Cases (%)		Overall Frequency
*SF3B1*	Non-AdvSM	2/309 (0.6) [12] 0/10 (0) [13] 0/44 (0) [29] 0/26 (0) [68] 0/6 (0) [85]	0.5%	BMM	1/90 (1) [12]		1%
				ISM	1/211 (0.5) [12] 0/44 (0) [29] 10/4 (0) [85]	0/3 (0) [13] 0/26 (0) [68]	0.3%
				SSM	0/8 (0) [12] 0/2 (0) [85]	0/7 (0) [13]	0%
	AdvSM	0/13 (0) [12] 3/24 (13) [13] 9/106 (9) [29] 18/305 (6) [32] 2/83 (2) [68] 1/13 (8) [85]	7%	ASM	0/9 (0) [12] 1/25 (4) [29] 0/1 (0) [85]	2/11 (18) [13] 0/3 (0) [68]	6%
				SM-AHN	0/4 (0) [12] 7/80 (9) [29] 1/12 (8) [85]	1/13 (8) [13] 2/72 (3) [68]	6%
				MCL	1/1 (100) [29]	0/8 (0) [68]	13%
*SRSF2*	Non-AdvSM	0/12 (0) [10] 0/309 (0) [12] 0/10 (0) [13] 0/44 (0) [29] 0/1 (0) [50] 0/26 (0) [68] 0/6 (0) [85] 7/530 (1) [97]	0.7%	BMM	0/90 (0) [12]		0%
				ISM	0/10 (0) [10] 0/3 (0) [13] 0/1 (0) [50] 0/4 (0) [85]	0/211 (0) [12] 0/44 (0) [29] 0/26 (0) [68]	0%
				SSM	0/2 (0) [10] 0/7 (0) [13]	0/8 (0) [12] 0/2 (0) [85]	0%
	AdvSM	14/27 (52) [10] 2/13 (15) [12] 3/24 (13) [13] 1/106 (1) [29] 120/329 (37) [32] 8/25 (32) [50] 31/83 (37) [68] 4/13 (31) [85] 79/210 (38) [97]	32%	ASM	0/1 (0) [10] 0/11 (0) [13] 1/2 (50) [50] 0/1 (0) [85]	1/9 (11) [12] 0/25 (0) [29] 0/3 (0) [68]	4%
				SM-AHN	13/23 (57) [10] 3/13 (23) [13] 7/21 (33) [50] 4/12 (33) [85]	1/4 (25) [12] 1/80 (1) [29] 31/72 (43) [68]	27%
				MCL	1/3 (33) [10] 0/2 (0) [50]	0/1 (0) [29] 0/8 (0) [68]	7%

Overall frequencies represent the weighted average of the percentage of patients with at least one mutation in that gene out of the total number of patients studied within the different cohorts for each subgroup of SM. Abbreviations: AdvSM: advanced systemic mastocytosis (SM); ASM: aggressive SM; BMM: bone marrow mastocytosis; ISM: indolent SM; MCL: mast cell leukaemia; Non-AdvSM: non-advanced SM; SM-AHN: SM with an associated haematological neoplasm; SSM: smouldering SM.

## Supplementary Materials

The following supporting information can be downloaded at: https://www.mdpi.com/article/10.3390/cancers14102487/s1, Table S1: Sporadic gene mutations reported in systemic mastocytosis patients; Table S2: Frequency of mutations involving genes other than KIT found to be sporadically mutated in systemic mastocytosis patients; Table S3: Frequency of mutations involving genes other than KIT in patients with systemic mastocytosis associated to another haematological neoplasm.
