# Supplementary material for: Comprehensive Analysis of Acquired Genetic Variants and Their Prognostic Impact in Systemic Mastocytosis
Source: Cancers (Basel). 2022 May 18;14(10):2487. doi: 10.3390/cancers14102487 (PMC9139197; doi:10.3390/cancers14102487)
Supplement: Supplementary file 1 [file cancers-14-02487-s001.zip › cancers-1690546-supplementary.pdf]

cancers MDPI

Supplemental Materials

Table S1. Sporadic gene mutations reported in systemic mastocytosis patients.

Gene	Exon	Gene Mutations			
CALR	**9**	**L367_fs* [29]**	R376C [29]		
CDH11	4	V168M [156]	P169L [13]		
	10	R477M [12]			
CILK1	2	G16S [13]			
CSF3R	17	M696T [29]	R698C [29]	E808K [29]	T829I [29]
EPHA7	11	T666I [12]	Q675P [12]		
	14	Q809R [13]			
ETNK1	5	N244S [177]	G245A [177]		
ETV6	5	H308Y [10]			
FLT3	13	V557I [29]			
IDH1	4	R132C [29]			
IDH2	4	R140Q [29,32]			
IKZF1	5	N159S [13]			
ITGA10	7	T224M [12]			
	9	Q335H [12]			
	11	F420L [13]			
	16	D665N [12]			
	18	S778T [12]			
	20	I843M [12]			
	25	I986M [12]			
	27	R1075* [12]			
	30	Q1167* [12]			
KAT6B	13	R871Q [12]			
	18	E1366_E1368* [13]	C1704F [13]		
NPM1	11	W288Lfs* [29]			
PIK3CD	4	V92M [12]			
	14	L598P [13]			
	20	R821C [12]			
	23	982W [12]			
ROS1	8	S252A [13]			
	19	G910V [12]			
	21	G1027D [12]			
	34	T1816P [12]			
	40	C2067F [12]			
	44	N2333K [13]			
SETBP1	4	D868N [29]	I871N [29]		
TP53	7	R248Q [29]			
	8	R282G [29]			
U2AF1	2	S34F [29,156]/Y [157]		P40R [32]	S43R [32]
	6	Q157P/R [29]			

*: Stop codon resulting in an incomplete protein.

Table S2. Frequency of mutations involving genes other than *KIT* found to be sporadically mutated in systemic mastocytosis patients.

Gene	SM Prognostic Subgroup	Mutated Cases/Total Cases (%)	Overall Frequency	WHO Subtype	Mutated Cases/ Total Cases (%)		Overall Frequency
CALR	Non-AdvSM	0/44 (0) [29]	**0%**	BMM			
				ISM	0/44 (0) [29]		**0%**
				SSM			
	AdvSM	2/106 (2) [29]	**2%**	ASM	1/25 (4) [29]		**4%**
				SM-AHN	1/80 (1) [29]		**1%**
				MCL	0/1 (0) [29]		**0%**
CDH11	Non-AdvSM	1/309 (0.3) [12]<br>0/10 (0) [13]	**0.3%**	BMM	0/90 (0) [12]		**0%**
				ISM	1/211 (0.5) [12]	0/3 (0) [13]	**0.5%**
				SSM	0/8 (0) [12]	0/7 (0) [13]	**0%**
	AdvSM	0/13 (0) [12]<br>1/24 (4) [13]	**3%**	ASM	0/9 (0) [12]	0/11 (0) [13]	**0%**
				SM-AHN	0/4 (0) [12]	1/13 (8) [13]	**6%**
				MCL			
CILK1	Non-AdvSM	0/309 (0) [12]<br>0/10 (0) [13]	**0%**	BMM	0/90 (0) [12]		**0%**
				ISM	0/211 (0) [12]	0/3 (0) [13]	**0%**
				SSM	0/8 (0) [12]	0/7 (0) [13]	**0%**
	AdvSM	0/13 (0) [12]<br>1/24 (4) [13]	**3%**	ASM	0/9 (0) [12]	0/11 (0) [13]	**0%**
				SM-AHN	0/4 (0) [12]	1/13 (8) [13]	**6%**
				MCL			
CSF3R	Non-AdvSM	0/44 (0) [29]	**0%**	BMM			
				ISM	0/44 (0) [29]		**0%**
				SSM			
	AdvSM	1/106 (1) [29]	**1%**	ASM	0/25 (0) [29]		**0%**
				SM-AHN	1/80 (1) [29]		**1%**
				MCL	0/1 (0) [29]		**0%**
EPHA7	Non-AdvSM	2/309 (0.6) [12]<br>0/10 (0) [13]	**0.6%**	BMM	1/90 (1) [12]		**1%**
				ISM	2/211 (0.9) [12]	0/3 (0) [13]	**0.9%**
				SSM	0/8 (0) [12]	0/7 (0) [13]	**0%**
	AdvSM	0/13 (0) [12]<br>1/24 (4) [13]	**3%**	ASM	0/9 (0) [12]	1/11 (9) [13]	**5%**
				SM-AHN	0/4 (0) [12]	0/13 (0) [13]	**0%**
				MCL			
ETNK1	Non-AdvSM	0/26 (0) [68]<br>0/36 (0) [179]	**0 %**	BMM			
				ISM	0/26 (0) [68]	0/36 (0) [179]	**0%**
				SSM			
	AdvSM	1/83 (1) [68]<br>5/46 (11) [179]	**5%**	ASM	0/3 (0) [68]	1/20 (5) [179]	**5%**
				SM-AHN	1/72 (1) [68]	4/26 (15) [179]	**5%**
				MCL	0/8 (0) [68]		**0%**
ETV6	Non-AdvSM	0/12 (0) [10]	**0%**	BMM			
				ISM	0/10 (0) [10]		**0%**
				SSM	0/2 (0) [10]		**0%**
	AdvSM	1/27 (4) [10]	**4%**	ASM	0/1 (0) [10]		**0%**
				SM-AHN	1/23 (4) [10]		**4%**
				MCL	0/3 (0) [10]		**0%**
FLT3	Non-AdvSM	0/6 (0) [13]<br>0/44 (0) [29]	**0%**	BMM			
				ISM	0/3 (0) [13]	0/44 (0) [29]	**0%**
				SSM	0/3 (0) [13]		
	AdvSM	0/14 (0) [13]<br>2/106 (2) [29]	**2%**	ASM	0/9 (0) [13]	0/25 (0) [29]	**0%**
				SM-AHN	0/5 (0) [13]	2/80 (2) [29]	**2%**
				MCL	0/1 (0) [29]		**0%**
IDH1/2	Non-AdvSM	0/6 (0) [13]<br>0/44 (0) [29]	**0%**	BMM			
				ISM	0/3 (0) [13]	0/44 (0) [29]	**0%**

		0/1 (0) [50]<br>0/26 (0) [68]<br>0/15 (0) [80]<br>0/6 (0) [85]			0/1 (0) [50]<br>0/15 (0) [80]	0/26 (0) [68]<br>0/4 (0) [85]	
				SSM	0/3 (0) [13]	0/2 (0) [85]	**0%**
	AdvSM	0/14 (0) [13]<br>4/106 (4) [29]<br>15/272 (6) [32]<br>3/25 (12) [50]<br>2/83 (2) [68]<br>0/10 (0) [80]<br>1/13 (8) [85]	**5%**	ASM	0/9 (0) [13]<br>0/2 (0) [50]<br>0/2 (0) [80]	1/25 (4) [29]<br>0/3 (0) [68]<br>0/1 (0) [85]	**2%**
				SM-AHN	0/5 (0) [13]<br>3/21 (14) [50]<br>0/8 (0) [80]	3/80 (4) [29]<br>2/72 (3) [68]<br>1/12 (8) [85]	**5%**
				MCL	0/1 (0) [29]<br>0/8 (0) [68]	0/2 (0) [50]	**0%**
IKZF1	Non-AdvSM	0/309 (0) [12]<br>0/10 (0) [13]<br>0/44 (0) [29]	**0%**	BMM	0/90 (0) [12]		**0%**
				ISM	0/211 (0) [12]<br>0/44 (0) [29]	0/3 (0) [13]	**0%**
				SSM	0/8 (0) [12]	0/7 (0) [13]	**0%**
	AdvSM	0/13 (0) [12]<br>2/24 (8) [13]<br>0/106 (0) [29]	**1%**	ASM	0/9 (0) [12]<br>0/25 (0) [29]	2/11 (18) [13]	**6%**
				SM-AHN	0/4 (0) [12]<br>0/80 (0) [29]	0/13 (0) [13]	**0%**
				MCL	0/1 (0) [29]		
ITGA10	Non-AdvSM	8/309 (3) [12]<br>1/10 (10) [13]	**3%**	BMM	2/90 (2) [12]		**2%**
				ISM	6/211 (3) [12]	0/3 (0) [13]	**3%**
				SSM	0/8 (0) [12]	1/7 (14) [13]	**7%**
	AdvSM	0/13 (0) [12]<br>0/24 (0) [13]	**0%**	ASM	0/9 (0) [12]	0/11 (0) [13]	**0%**
				SM-AHN	0/4 (0) [12]	0/13 (0) [13]	**0%**
				MCL			
KAT6B	Non-AdvSM	1/309 (0.3) [12]<br>0/10 (0) [13]	**0.3%**	BMM	1/90 (1) [12]		**1%**
				ISM	0/211 (0) [12]	0/3 (0) [13]	**0%**
				SSM	0/8 (0) [12]	0/7 (0) [13]	**0%**
	AdvSM	0/13 (0) [12]<br>2/24 (8) [13]	**5%**	ASM	0/9 (0) [12]	1/11 (9) [13]	**5%**
				SM-AHN	0/4 (0) [12]	1/13 (8) [13]	**6%**
				MCL			
NPM1	Non-AdvSM	0/6 (0) [13]<br>0/44 (0) [29]<br>0/26 (0) [68]	**0%**	BMM			
				ISM	0/3 (0) [13]<br>0/44 (0) [29]<br>0/26 (0) [68]		**0%**
				SSM	0/3 (0) [13]		**0%**
	AdvSM	0/14 (0) [13]<br>1/106 (1) [29]<br>4/83 (5) [68]	**3%**	ASM	0/9 (0) [13]<br>0/3 (0) [68]	0/25 (0) [29]	**0%**
				SM-AHN	0/5 (0) [13]<br>4/72 (6) [68]	1/80 (1) [29]	**4%**
				MCL	0/1 (0) [29]	0/8 (0) [68]	**0%**
PIK3CD	Non-AdvSM	3/309 (1) [12]<br>0/10 (0) [13]	**1%**	BMM	2/90 (2) [12]		**2%**
				ISM	1/211 (0.5) [12]	0/3 (0) [13]	**0.5%**
				SSM	0/8 (0) [12]	0/7 (0) [13]	**0%**
	AdvSM	0/13 (0) [12]<br>1/24 (4) [13]	**3%**	ASM	0/9 (0) [12]	1/11 (9) [13]	**5%**
				SM-AHN	0/4 (0) [12]	0/13 (0) [13]	**0%**
				MCL			
ROS1	Non-AdvSM	4/309 (1) [12]<br>1/10 (10) [13]	**2%**	BMM	2/90 (2) [12]		**2%**
				ISM	2/211 (1) [12]	0/3 (0) [13]	**1%**
				SSM	0/8 (0) [12]	1/7 (14) [13]	**7%**
	AdvSM	0/13 (0) [12]<br>1/24 (4) [13]	**3%**	ASM	0/9 (0) [12]	0/11 (0) [13]	**0%**
				SM-AHN	0/4 (0) [12]	1/13 (8) [13]	**6%**
				MCL			

SETBP1	Non-AdvSM	1/12 (8) [10]<br>0/44 (0) [29]<br>0/26 (0) [68]	**1%**	BMM			
				ISM	1/10 (10) [10]<br>0/26 (0) [68]	0/44 (0) [29]	**1%**
				SSM	0/2 (0) [10]		**0%**
	AdvSM	0/27 (0) [10]<br>4/106 (4) [29]<br>2/83 (2) [68]<br>2/19 (11) [90]	**3%**	ASM	0/1 (0) [10]<br>0/3 (0) [68]	1/25 (4) [29]<br>1/6 (17) [90]	**6%**
				SM-AHN	0/23 (0) [10]<br>2/72 (3) [68]	3/80 (4) [29]<br>1/13 (8) [90]	**3%**
				MCL	0/3 (0) [10]<br>0/8 (0) [68]	0/1 (0) [29]	**0%**
TP53	Non-AdvSM	0/44 (0) [29]<br>0/6 (0) [13]	**0%**	BMM			
				ISM	0/3 (0) [13]	0/44 (0) [29]	**0%**
				SSM	0/3 (0) [13]		**0%**
	AdvSM	0/14 (0) [13]<br>3/106 (3) [29]<br>1/16 (6) [68]	**3%**	ASM	0/9 (0) [13]	0/25 (0) [29]	**0%**
				SM-AHN	0/5 (0) [13]<br>1/16 (6) [68]	3/80 (4) [29]	**4%**
				MCL	0/1 (0) [29]		**0%**
U2AF1	Non-AdvSM	1/11 (9) [10]<br>0/44 (0) [29]<br>0/1 (0) [50]<br>0/26 (0) [68]<br>0/6 (0) [85]	**1%**	BMM			
				ISM	1/10 (10) [10]<br>0/1 (0) [50]<br>0/4 (0) [85]	0/44 (0) [29]<br>0/26 (0) [68]	**1%**
				SSM	0/1 (0) [10]	0/2 (0) [85]	**0%**
	AdvSM	1/27 (4) [10]<br>6/106 (6) [29]<br>2/25 (8) [50]<br>4/83 (5) [68]<br>1/13 (8) [85]	**6%**	ASM	0/1 (0) [10]<br>1/2 (50) [50]<br>0/1 (0) [85]	0/25 (0) [29]<br>0/3 (0) [68]	**3%**
				SM-AHN	1/23 (5) [10]<br>1/21 (5) [50]<br>1/12 (8) [85]	6/80 (8) [29]<br>4/72 (6) [68]	**6%**
				MCL	0/3 (0) [10]<br>0/2 (0) [50]	0/1 (0) [29]<br>0/8 (0) [68]	**0%**

Overall frequencies represent the weighted average of the percentage of patients with at least one mutation in that gene out of the total number of patients studied within the different cohorts for each SM subgroup. Abbreviations: AdvSM: advanced systemic mastocytosis (SM); ASM: aggressive SM; BMM: bone marrow mastocytosis; ISM: indolent SM; MCL: mast cell leukaemia; Non-AdvSM: non-advanced SM; SM-AHN: SM with an associated haematological neoplasm; SSM: smouldering SM.

Table S3. Frequency of mutations involving genes other than *KIT* in patients with systemic mastocytosis with an associated haematological neoplasm.

	AHN Subtype							
	MDS		MPN		CMML		AML	
**Gene**	**Mutated Cases/ Total Cases**	**Overall Frequency**	**Mutated Cases/ Total Cases**	**Overall Frequency**	**Mutated Cases/ Total Cases**	**Overall Frequency**	**Mutated Cases/ Total Cases**	**Overall Frequency**
ASXL1	0/1 [10]; 0/3 [13]; 2/8 [68]; 3/12 [81]; 2/3 [85]	**26%**	4*/12 [10]; 4*/28 [68]; 0/3 [85]; 2/13 [81]	**18%**	3/7 [10]; 1/7 [81]; 3/6 [85]	**35%**	1/3 [10]; 1/6 [13]; 3/9 [68]; 1/3 [81]	**29%**
CBL	0/1 [10]; 0/9 [51]; 1/8 [68]; 1/3 [85]	**10%**	5*/12 [10]; 0/5 [51]; 0/3 [85]; 4*/28 [68]	**19%**	1/7 [10]; 0/5 [51]; 4*/26 [68]; 1/6 [85]	**13%**	0/3 [10]; 0/3 [51]; 0/9 [68]	**0%**
DNMT3A	0/8 [68]	**0%**	1/28 [68]	**4%**	0/26 [68]	**0%**	0/9 [68]	**0%**
EZH2	0/1 [10]; 1/3 [13]; 1/8 [68]; 0/3 [85]	**13%**	2/12 [10]; 1/28 [68]; 1/3 [85]	**9%**	0/7 [10]; 0/26 [68]; 1/6 [85]	**3%**	0/3 [10]; 1/6 [13]; 0/9 [68]	**6%**
JAK2	0/1 [10]; 1/9 [51]; 0/8 [68]; 0/3 [85]	**5%**	2*/12 [10]; 2/5 [51]; 10*/28 [68]; 1/3 [85]	**31%**	0/7 [10]; 0/5 [51]; 1/26 [68]; 0/6 [85]	**2%**	0/3 [10]; 0/3 [51]; 0/9 [68]	**0%**
K/NRAS	0/1 [10]; 0/9 [51]; 1*/8 [68]; 0/3 [85]	**5%**	3/12 [10]; 1/5 [51]; 2*/28 [68]; 0/3 [85]	**13%**	0/5 [51]; 3*/26 [68]; 0/6 [85]	**8%**	1/3 [10]; 0/3 [51]; 2*/9 [68]	**20%**
RUNX1	0/1 [10]; 1/3 [13]; 1/8 [68]; 0/3 [85]	**13%**	3/12 [10]; 3*/28 [68]; 0/2 [85]	**14%**	2/7 [10]; 3/26 [68]; 1/6 [85]	**15%**	2/3 [10]; 3/6 [13]; 3*/9 [68]	**44%**
SF3B1	1/3 [13]; 1/8 [68]; 1/3 [85]	**21%**	0/28 [68]; 0/3 [85]	**0%**	0/26 [68]; 0/6 [85]	**0%**	0/6 [13]; 0/9 [68]	**0%**
SRSF2	0/1 [10]; 2/3 [13]; 2*/8 [68]; 0/3 [85]	**27%**	6*/12 [10]; 10**/28 [68]; 0/3 [85]	**37%**	6/7 [10]; 14**/26 [68]; 3/6 [85]	**59%**	1/3 [10]; 1/6 [13]; 2/9 [68]	**22%**
TET2	0/1 [10]; 1/3 [13]; 4/9 [51]; 1/8 [68]; 5/14 [81]; 0/3 [85]; 2/6 [88]	**30%**	8*/12 [10]; 0/5 [51]; 1/12 [81]; 11**/28 [68]; 2/6 [88]; 1/3 [85]	**35%**	6/7 [10]; 3/5 [51]; 4/7 [81]; 18**/26 [68]; 5/6 [85]; 4/8 [88]	**68%**	1/3 [10]; 0/6 [13]; 2/3 [51]; 2/9 [68]; 1/3 [81]	**25%**

Overall frequencies represent the weighted average of the percentage of patients with at least one mutation in that gene out of the total number of patients studied within the different cohorts for each AHN subtype. *1 case of mutated mast cell leukaemia (MCL) within the mast cell component of the disease; **2 cases of mutated MCL-AHN. Abbreviations: AHN: associated haematological neoplasm; AML: acute myeloid leukaemia; CMML: chronic myelomonocytic leukaemia; MDS: myelodysplastic syndromes; MPN: myeloproliferative neoplasms.
